# “Vaccinate, Do Not Hesitate!”. Vaccination Readiness against COVID-19 among Polish Nursing Undergraduate Students: A National Cross-Sectional Survey

**DOI:** 10.3390/vaccines9091029

**Published:** 2021-09-16

**Authors:** Joanna Gotlib, Tomasz Sobierajski, Mariusz Jaworski, Dominik Wawrzuta, Ewa Borowiak, Beata Dobrowolska, Danuta Dyk, Aleksandra Gaworska-Krzemińska, Elżbieta Grochans, Maria Kózka, Halina Kulik, Jolanta Lewko, Grażyna Nowak-Starz, Małgorzata Wojciechowska, Izabella Uchmanowicz, Mariusz Panczyk

**Affiliations:** 1 Department of Education and Health Sciences Research, Medical University of Warsaw, 02-091 Warsaw, Poland; joanna.gotlib@wum.edu.pl (J.G.); mariusz.jaworski@wum.edu.pl (M.J.); mariusz.panczyk@wum.edu.pl (M.P.); 2 Faculty of Applied Social Sciences and Resocialization, University of Warsaw, 00-325 Warsaw, Poland; tomasz.sobierajski@uw.edu.pl; 3 Department of Conservative Nursing, Medical University of Lodz, 90-926 Lodz, Poland; ewa.borowiak@umed.lodz.pl; 4 Department of Management in Nursing, Medical University of Lublin, 20-109 Lublin, Poland; beata.dobrowolska@umlub.pl; 5 Department of Anaesthesiology and Intensive Care Nursing, Medical University in Poznan, 61-841 Poznan, Poland; dyk@ump.edu.pl; 6 Department of Nursing Management, Medical University in Gdansk, 80-803 Gdansk, Poland; aleksandra.gaworska-krzeminska@gumed.edu.pl; 7 Department of Nursing, Pomeranian Medical University in Szczecin, 70-456 Szczecin, Poland; grochans@pum.edu.pl; 8 Department of Clinical Nursing, Jagiellonian University Medical College, 31-007 Krakow, Poland; makozka@cm-uj.krakow.pl; 9 Department of Propaedeutics of Nursing, Medical University in Katowice, 41-100 Katowice, Poland; hkulik@sum.edu.pl; 10 Department of Primary Health Care, Faculty of Health Sciences, Medical University in Bialystok, 15-950 Bialystok, Poland; jola.lewko@wp.pl; 11 Institute of Public Health, Jan Kochanowski University Medical College, 25-406 Kielce, Poland; gnowakstarz@ujk.edu.pl; 12 Department of Nursing, University of Physical Education in Warsaw, 00-968 Warsaw, Poland; malgorzata.wojciechowska@awf.edu.pl; 13 Faculty of Health Sciences, Wroclaw Medical University, 50-141 Wroclaw, Poland; izabella.uchmanowicz@umed.wroc.pl

**Keywords:** COVID-19 vaccine, nursing students, level of knowledge, readiness, vaccine hesitancy

## Abstract

COVID-19 vaccination raises numerous concerns among the public, and also among medical personnel including nurses. As nurses play a crucial role in the process of vaccination, it is important to recognize the attitudes of students of nursing, nurses in spe, toward COVID-19 vaccination, as well as to define the factors influencing students’ pro-vaccine choices. The study was conducted between March and April 2021 at all medical universities in Poland educating nurses in spe. The study included 793 first-degree students from 12 universities. The results revealed that the vast majority of students of nursing (77.2%) were vaccinated against COVID-19, as 61.2% received an mRNA vaccine and 16% a viral vector vaccine. Every other person in the non-vaccinated group declared their intention to get a vaccination. A trend was observed whereby people co-living with persons from the risk group, who are at risk of a severe form of COVID-19, showed greater willingness to get a vaccine. The study results identified the role of universities in increasing the vaccination rate among students, both in terms of education about vaccinations and in shaping pro-vaccine attitudes among students, as well as organizing vaccinations on university campuses to facilitate the process.

## 1. Introduction

It has been accepted worldwide that in the absence of an effective treatment for the SARS-CoV-2 virus and the consequential COVID-19, only the rapid vaccination of an adequate number of people and the establishment of herd immunity will inhibit the spread of consecutive waves of infection, limit the spread of the pandemic, and increase the chance of its termination [1].

The first COVID-19 vaccination approved for regular use was BNT162b2 (Comirnaty^®^, Amsterdam, The Netherlands; BioNTech/Pfizer). It was approved by the National Health Service of Great Britain on 7 December 2020 [2]. Since then, vaccinations organized by governments have been implemented in 174 countries, using 15 vaccines, approved for use by at least one regulator [3]. According to the most recent data (4 July 2021), 884,440,000 people have been fully vaccinated worldwide [4], mostly in China and the USA. Vaccinations against SARS-CoV-2 in Poland started on 27 December 2020. As of 4 July 2021, 17,114,524 people had received their first dose, and 12,909,625 Poles were fully vaccinated [5].

Despite different regulations in each country regarding the course and schedule of mass vaccinations, the majority of countries vaccinated medical personnel and the elderly first. In Poland, the first group vaccinated, called Group Zero, comprised the medical and non-medical personnel of medical facilities, pharmacies, and nursing homes, as well as students of all faculties at medical universities. The process started in the majority of Polish medical universities on 4 January 2021 [6].

From the start of global vaccinations, the attitudes of societies toward COVID-19 vaccination have varied from enthusiasm to skepticism [7,8]. In many countries, attitudes of unwillingness have been present among medical personnel, e.g., doctors and nurses [9]. The arguments most often employed against getting a vaccine are the safety of the vaccine and its adverse events, raised by the rapid completion of clinical trials and the introduction of the vaccines onto the market, as well as insecurity related to the length of the personal immune response provided by the vaccine [10,11].

Decisions about vaccination and uncertainty related to its efficiency are linked to emotional tension (e.g., stress) [12] and certain personality traits (e.g., neuroticism, conscientiousness, and locus of control) [13]. Stress is vital to decision making as it magnifies cognitive distortions such as attention selectivity. People facing a difficult decision, such as whether or not to be vaccinated, experience strong emotional tension and must address the cost–benefit balance. However, they pay particular attention to the profit. By doing so, they understate the significance of negative consequences. It should be noted that the evaluation of profits and losses is always based on an individual’s value system [12]. In this view, the beliefs of an individual, e.g., their altruistic beliefs, may play an important role [13]. Additionally, in line with the fuzzy-trace theory, decisions about vaccination may be dichotomous, involving choosing between well-being and feeling unwell. People are prone to take part in activities affecting their well-being. The current emotional condition of a person, as well as the individual cognitive evaluation of personal well-being, are significant in this context [14].

The majority of the available research results show that insecurity about getting a vaccination does not only affect medical personnel [15]. A large group of students with medical degrees have also been shown to be uncertain about getting the vaccination, or only consider the possibility once the vaccination is available [16,17,18,19,20]. Therefore, we aimed to investigate the attitudes of the students of nursing programs at medical universities in Poland toward vaccinations against COVID-19, and to identify factors that influence their decision for or against being vaccinated. We believe that this study is extremely important, as it addresses the decision pathway(s) of nursing students at medical universities throughout Poland in the context of prioritizing vaccinations for this group, along with medical personnel.

### Aim of the Study

The purpose of this study was to describe the levels and sources of knowledge that undergraduate students of nursing possess, and their attitudes toward and willingness to receive a COVID-19 vaccine since it became available in Poland (January 2021).

## 2. Materials and Methods

### 2.1. Design and Setting

This national cross-sectional, online survey study was conducted from March to April 2021. Twelve universities that run an undergraduate nursing program in Poland participated in the study.

### 2.2. Local Context

Participation of students of nursing in the Polish national vaccination campaign was voluntary. Volunteers booked their vaccination time online. After logging in, access was given to an individual account with a calendar and a list of vacant times (vaccination point, date, and time of the appointment). The booking was confirmed by e-mail. A day before the vaccination, a reminder was sent. The patient could cancel or reschedule the appointment at the vaccination point.

### 2.3. Sample Size

The target population was 7000 Polish undergraduate nursing students. The total potential number of respondents was 4700 students from 12 universities. Data were, however, only received from 793 students. The sample was representative of a broader spectrum of Polish students of nursing. With this sample size and the number of students of nursing in Poland (*N* = 7000), the error margin was 2.00% (95% confidence level and 0.75 proportion).

### 2.4. Participants

Students of the three-year Bachelor’s nursing degree qualified for this study. Study participants were recruited by the employees of 12 universities who agreed to participate as study coordinators. Every coordinator was trained in terms of the aim of the study, the means of distribution of the research tools, and the principles of data collection monitoring in the study. Altogether, 850 students agreed to participate in this study, and a full data package was collected from 793 of them.

### 2.5. Instrument

The questionnaire used herein was originally created for this study. The survey’s development was based on the current literature about COVID-19 and our previous research [21]. The questionnaire consisted of three sections, namely (1) demographics, (2) motivations and attitudes toward COVID-19 vaccination, and (3) vaccine information sources. The questionnaire used the following types of questions: Likert scale, closed-ended, semi-open, and open-ended questions. Completion of the online questionnaire took approximately 25 min.

The 18 items in the survey included the following demographics: Year and level of program, sex, age, place of residence, chronic illnesses, flu vaccine uptake, professional plans, internship in wards handling COVID-19 patients, information on COVID-19 exposure and/or infection (own or in the immediate family), course of the disease, information on getting vaccinated, place of vaccination, and the type and incidence of adverse events of the vaccine.

The questions about motivation firstly considered to what degree the concern for oneself and one’s relatives contributed to the decision regarding vaccination. The four items in the survey included questions regarding attitudes toward COVID-19 vaccination. In the last section of the questionnaire, the questions addressed the frequency of accessing vaccine information sources (a total of 13 items).

The pilot survey was completed by 20 nursing faculty members and 20 students of nursing (Delphi study). Revisions were made to improve clarity. The survey was also available in English upon request.

### 2.6. Data Collection

The questionnaire was distributed with the aid of the Lime Survey web platform. The link to the survey was shared with 11 coordinators at the participating universities. The mode of survey distribution was determined by the limited chance for direct contact with the respondents, linked to restrictions introduced by the Minister of Health related to the COVID-19 pandemic. Thus, online study was the recommended approach, enabling quick access to the study group and ensuring security [22,23].

### 2.7. Ethical Considerations

The study protocol was approved by the University’s Ethics Committee (IRB approval no. KB/76/2021).

Before entering the study, participants were informed of their anonymity and the confidentiality of the data collected. No personal data, including computer IP, were collected. To ensure the anonymous nature of the questionnaire, it was not possible to track sensitive personal data.

### 2.8. Data Analysis

Quantitative and categorical variables were derived using descriptive statistics. As regards the quantitative variables, the following measures were determined: Central tendency (mean (M)), dispersion (standard deviation (SD)). For categorical variables, the following measures were determined: Number (N) and frequency (%).

Cross-tabulation and chi-squared tests were used to evaluate the impacts of the selected factors on pro-vaccine decisions. For the comparison of the respondents’ levels of trust in the vaccines depending on their willingness to immunize, analysis of variance (ANOVA) was used.

All calculations were performed with STATISTICATM 13.3 software (TIBCO Software, Palo Alto, CA, USA). For all analyses, a *p*-value of <0.05 was considered statistically significant.

## 3. Results

### 3.1. Sample Characteristic

In total, 793 Polish undergraduate nursing students participated in this study. The average age of study participants was 22.4 years (SD = 5.04). The majority of the respondents were second-year students (*N* = 335, 42.2%), and women comprised the vast majority (*N* = 720, 90.8%), which is consistent with the average gender distribution in the nursing faculties in Poland. The sample group was ethnically homogeneous. The selected profile of the studied group is presented in Table 1.

### 3.2. Willingness to Get a COVID-19 Vaccination

The vast majority of the respondents had already received a COVID-19 vaccination (*N* = 612, 77.2%), usually with an mRNA vaccine (*N* = 485, 61.2%) on the university campuses (*N* = 502, 63.3%). In less than one third of those vaccinated, adverse effects occurred (*N* = 225, 36.8%). A detailed summary of responses to COVID-19 vaccination is presented in Table 2.

In the unvaccinated group (*N* = 179), every third student (*N* = 59, 32.9%) declared that they would not undergo vaccination (replied “definitely not” or “rather not”). One in every five students from the unvaccinated group (*N* = 35, 19.5%) had not yet decided, 38% declared that they would be vaccinated (replied “definitely yes” or “rather yes”), and one in every 10 (*N* = 19, 10.6%) declared that they could not be vaccinated due to health problems.

Based on the given reasons for or against vaccination, evaluated on a seven-point scale (from 0 to 6), it was revealed that the decision for or against vaccination was made mainly with the relatives’ health in mind (M = 5.30, SD = 1.32). The care for one’s own health held slightly lower significance (M = 4.28, SD = 1.73).

### 3.3. Factors That Influence Vaccination Decisions

The factors linked to the willingness to get a COVID-19 vaccination were analyzed in a group of respondents who were not vaccinated at the time of the study and did not have any medical restrictions (*N* = 162). The analysis of potential factors that could influence the decision for vaccination did not reveal statistical significance (Table 3). In terms of the greater willingness to be vaccinated shown by students living with a person/people particularly vulnerable to severe COVID-19, a statistical trend was observed (*χ*^2^ = 5.714, *p* = 0.057).

**Figure 1:**
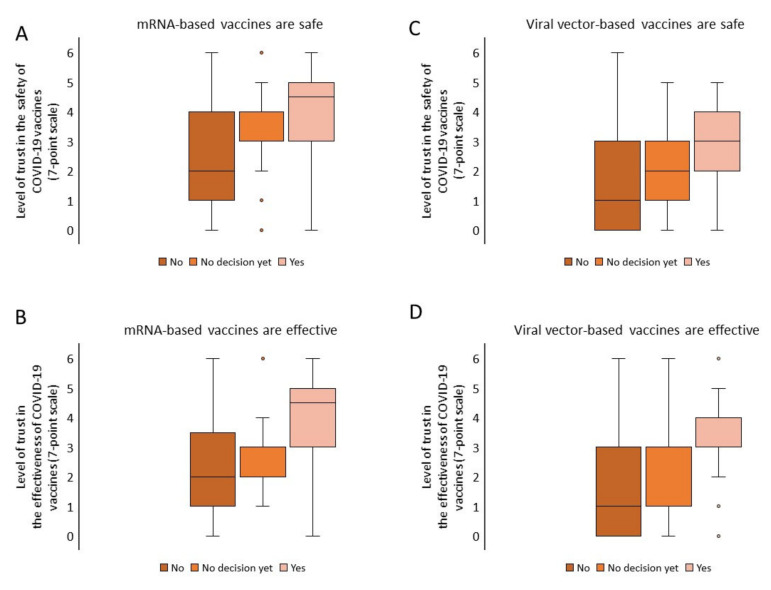
Perception of the safety and efficacy of COVID-19 vaccines in groups with different levels of willingness to be vaccinated. Analysis of the mean level of trust in groups with different levels of willingness to be vaccinated (ANOVA): (**A**) *F*_(2, 153)_ = 21.415, *p* < 0.001; (**B**) *F*_(2, 153)_ = 25.902, *p* < 0.001; (**C**) *F*_(2, 153)_ = 15.840, *p* < 0.001; (**D**) *F*_(2, 153)_ = 17.349, *p* < 0.001.

However, the level of trust in the safety and effectiveness of the COVID-19 vaccines, evaluated on a seven-point scale (from 0 to 6), was significantly higher among students expressing their willingness to be vaccinated compared to those who did not make such a decision or refused to be vaccinated (Figure 1).

### 3.4. Vaccine Information Sources

In total, 40.6% of the respondents (*N* = 322) indicated that the university provided access to state-of-the-art knowledge on vaccinations, whereas 35.6% (*N* = 282) stated they had no access to such information. The remaining 23.8% (*N* = 189) of the students were not aware of any existing sources of knowledge on vaccinations at their university.

**Figure 2:**
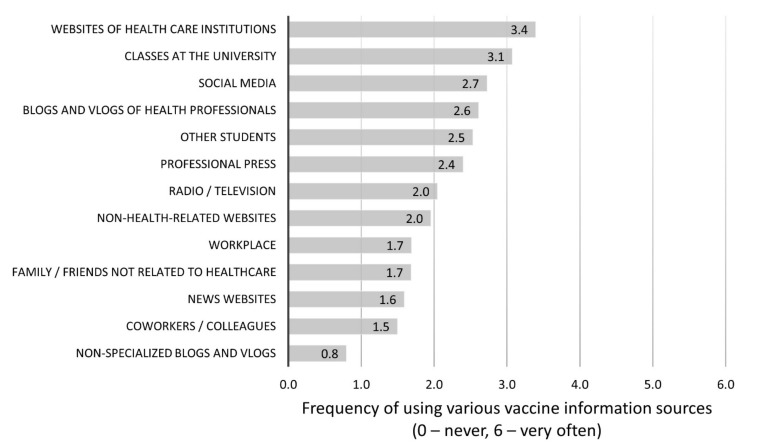
Average frequency of using various vaccine information sources (0—never; 6—very often).

Out of the various sources of information about vaccinations, the responders most often referred to the web pages of institutions linked to healthcare (M = 3.40, SD = 1.89), university classes (M = 3.07, SD = 1.86), and social media (M = 2.73, SD = 1.96) (Figure 2).

## 4. Discussion

To the best of our knowledge, this is the first study describing the willingness to be vaccinated, as well as the motives and attitudes of a representative nationwide sample of nursing students in the context of COVID-19. Attitudes were analyzed at the time of the vaccine becoming available for students upon a vaccination campaign being organized by the students’ universities. Earlier publications have presented research results of studies conducted at only one American university in New Jersey, where out of 457 college students (enrolled in the Spring 2021 semester), 23% of respondents (*n* = 105) reported already being vaccinated [24]. The remaining studies addressed hypothetical attitudes of students toward COVID-19 vaccination, at a time when it was not available for students, in the USA [16], Albania, Greece, Cyprus, Spain, the Czech Republic, Kosovo, Italy [17], India [25], Pakistan [26], Saudi Arabia [27], and China [28].

### 4.1. The Role of Shaping Positive Attitudes toward Vaccinations among Nurses

The role of nurses in convincing people to vaccinate against COVID-19 is important, as nurses, in contrast to physicians or paramedics, spend most of their time in direct contact with a patient, carrying out nursing care, the majority of which consists of health education and promotion. Additionally, in many countries, nursing is one of the most publicly trusted professions (third place in Poland) [29]. As one of the most trusted professions, nurses play a decisive role in counseling patients about the risks of COVID-19. One of the key factors that may significantly affect the promotion of vaccination against COVID-19 is the presence of positive attitudes toward vaccination among nurses. The shaping of these attitudes should start during university studies. Students of nursing are a leading group in the worldwide COVID-19 vaccination campaign. In Poland, in April 2021, this role was recognized, as nursing students became one of the first groups eligible for COVID-19 vaccinations [30].

### 4.2. Factors Potentially Influencing Getting Vaccinated among Students of Nursing

In the studied group of Polish nursing students, the vast majority (77.2%) decided to get vaccinated against COVID-19 at the earliest possible opportunity, usually at their university (63.3%). These results are in line with those from the literature; most students who declared their willingness to be vaccinated came from countries of the Middle and Far East—China (76.3% and 57%) [28,31], Saudi Arabia (77.8%) [27], or Pakistan (86.2%) [26,32]. This result is likely an effect of the large scale of the COVID-19 pandemic in China, which was reported by Wang et al. [28] in their work expounding the extended protection motivation theory. In the remaining available European and American publications, a large number of students did not declare a hypothetical willingness to vaccinate—only 43.8% of vaccinated students were reported in studies conducted in seven European countries [17], and 45.3% were reported in the study of Manning [16]. However, in Poland, in the first few months of the COVID-19 vaccination program, three out of four nursing students took their first vaccine dose, and only 7.4% declared that they did not wish to be vaccinated. These results are important, as the polls conducted in a representative group of Poles indicate that among the youngest adult respondents aged 18–24 years (the age of the majority of student respondents), only 37% declared a willingness to be vaccinated [33].

Several factors may impact the positive attitudes of Polish nursing students toward COVID-19 vaccination. Easy access to vaccines seems the primary one. In the case of students of medical universities, including students of nursing, the whole process of vaccination was organized and supervised by the universities. The students were only required to e-mail their willingness to be vaccinated to the proper unit responsible for the organization of vaccinations at the university. In a reply, students received information with a time and place for the vaccination, organized at the university campus. This hypothesis is additionally supported by a comparison with the willingness to get a flu vaccine, which is an optional vaccine in Poland, but it is provided by an autonomous organization.

In the studied group, the vast majority (83.1%) did not get a flu vaccine. Thus, the additional effort required to be vaccinated may be one of the key factors determining the unwillingness to be vaccinated against flu among the students of nursing.

The psychological aspect of the organization of vaccinations, related to studies on the psychology of stress [12] and the fuzzy-trace theory [14], may be a substantial factor contributing to the high vaccination rate against COVID-19 in the respondents. The conclusion of previous studies suggest that, when experiencing strong negative emotions, an individual will take steps self-rated as beneficial and positively impacting their well-being [12,14]. Therefore, the organization of vaccinations at the university could have been evaluated positively by students in a stressful pandemic situation. The cognitive evaluation of the profit and loss must be subjective and individualized, and if doubt arises or the profit–loss balance is negative, motivation for vaccination will reduce [12].

Another factor that could potentially have positively influenced attitudes toward vaccination was the willingness to complete studies. Students of nursing (particularly the ones in their last year) are obliged to undertake a clinical internship at patients’ bedsides. This is part of the curriculum, and selected clinical units demand that students are vaccinated before starting classes in hospital wards where patients with impaired immunity are treated (oncological, transplantation, pediatric, etc.). It must be noted, however, that students are not forced, but only encouraged to be vaccinated.

Personal experiences and indirect experiences with COVID-19 could be a significant factor influencing a large proportion of the vaccinated, as nearly every fourth person (22.8%) admitted that a person in their immediate environment experienced a severe or very severe course of COVID-19. Therefore, by vaccinating, they protected themselves from developing the illness.

Another issue influencing vaccination could have been that the majority of the students resided with relatives/friends, including 17.8% with seniors and 29.8% with people from the COVID-19 risk group. By being vaccinated, the students were assured that they would be able to restrict virus transmission among their relatives. This thesis is further supported by the analysis of the students’ responses to the question about their vaccination motives, which did not differ from those presented in the literature [16]. In our group, students indicated their concern for their relatives’ health and, to a lesser degree, for their health, as the two most significant factors motivating them to be vaccinated. These results are in line with results reported elsewhere [16]. In the study of Manning et al., 68.9% of American students declared their willingness to be vaccinated on the grounds of protecting others, and 65.5% on the grounds of protecting themselves [16].

One of the factors that could increase chances of vaccination is the availability of reliable information, such as that based on research findings related to COVID-19 vaccines. The available research publications confirmed that a high level of knowledge about pandemics manifested a greater prevalence of approval for pandemic restrictions, as well as greater adherence to wearing masks, more frequent hand disinfection, avoidance of large gatherings, and maintaining distance from people [34].

The results regarding the sources of knowledge about COVID-19 vaccines amongst the group of respondents confirm the hypothesis that the web pages of healthcare institutions and university classes are most commonly used by students. Polish students mentioned social media third, while students from Europe [17], India [25], and the USA [16] pointed toward Instagram, Snapchat, and TikTok as their most-used sources of knowledge on pandemics and COVID-19 vaccinations. We think that the different results may be due to the study being conducted in March 2021, exactly a year after the pandemic’s outbreak. At this time, reliable information on COVID-19 was already available for students and had been presented during their university classes. The only other group of students who used the official web pages of the Ministry of Health in the initial stage of the pandemic (June 2020) was from Saudi Arabia (80.0%), and social media was indicated by only 20% of respondents [27]. According to the authors, a significant factor in this case conditioning the students’ answers may have been the difference between cultural circumstances. The latest research results show that deriving information on COVID-19 from social media alone, in contrast to using mainstream media, clearly impacts a belief in conspiracy theories and anti-vaccination attitudes [35].

In summary, easy access to the vaccine, the organization of vaccination at the university, experiences with relatives’ illnesses, and the presence of reliable information on COVID-19 communicated during university classes significantly influenced students’ willingness to get vaccinated against COVID-19.

### 4.3. Strengths and Limitations of this Study

In our study, the questionnaire was developed based on the information about COVID-19 available on the WHO, the Polish National Institute of Public Health, and the Ministry of Health websites, with validation performed to increase the reliability of the study. Furthermore, this study embraced a representative group of students from 12 Polish universities, which is another strength. However, the study has some limitations that should not be ignored. First, as this was an online cross-sectional survey, there is a chance of bias in the information. Furthermore, self-reported questionnaires can often result in information bias. Moreover, even though the examined nursing students were a representative group, there is a risk that only students supporting vaccination and those vaccinated participated in the survey of knowledge and attitudes toward COVID-19 vaccines, while anti-vaccinationists likely would not have participated. However, to the best of our knowledge, these are the first globally available research results addressing the attitudes of nursing students toward vaccines when the organizational responsibility is held by their home universities.

## 5. Conclusions

The presented results demonstrated that the manner in which the vaccination campaign is organized and the opportunity to be vaccinated at their university campus without the need to organize it on their own may significantly positively influence the attitudes of nursing students toward COVID-19 vaccinations, as well as increase their willingness to be vaccinated. Moreover, such organizations may be greatly beneficial in the context of psychological tension (stress) and increasing vaccination efficiency.

The students learned about vaccinations from a wide variety of sources. This suggests that information about vaccinations should be clear, understandable, and easily accessible. Various distribution channels for this information should be used, not only the standard ones (e.g., websites of healthcare institutions or classes at the university), but also social media.

These results could be very useful for policymakers responsible for the organization of vaccinations for medical students all over the world, and for all of the policymakers developing strategies to promote vaccination against COVID-19.

## Figures and Tables

**Table 1:** Characteristics of the study group (*N* = 793).

Department of Nursing, *N* (%)	
Pomeranian Medical University	241 (30.4)
Medical University of Warsaw	113 (14.2)
Medical University of Gdańsk	86 (10.8)
Jagiellonian University Medical College	65 (8.2)
Poznan University of Medical Sciences	61 (7.7)
Medical University of Lodz	46 (5.8)
Wroclaw Medical University	40 (5.0)
Medical University of Białystok	36 (4.5)
Medical University of Lublin	35 (4.4)
Medical University of Silesia	33 (4.2)
Jan Kochanowski University Medical College	20 (2.5)
University of Physical Education in Warsaw	17 (2.1)
Year of study, *N* (%)	
1	316 (39.8)
2	335 (42.2)
3	142 (17.9)
Gender, *N* (%)	
Female	720 (90.8)
Male	60 (7.6)
Refusal to answer	13 (1.6)
Age (years)	
M ± SD	22.4 ± 5.04
Residence, *N* (%)	
alone	100 (12.6)
with relatives/family/friends (excl. seniors)	552 (69.6)
with relatives/family/friends (incl. seniors)	141 (17.8)
Residence with a person from the COVID-19 risk group, *N* **(%)**	
Yes	236 (29.8)
No	557 (70.2)
Developing COVID-19 in a relative, *N* (%)	
Yes, severe or very severe	181 (22.8)
Yes, rather mild	390 (49.2)
No	171 (21.6)
Don’t know	51 (6.4)
Developing COVID-19, *N* (%)	
Yes (severe infection symptoms)	23 (2.9)
Yes (mild infection symptoms)	70 (8.8)
Yes (no infection symptoms)	9 (1.1)
Probably (no test confirmation)	173 (21.8)
No	337 (42.5)
Don’t know	181 (22.8)

M, mean; SD, standard deviation.

**Table 2:** COVID-19 vaccination: Implementation, procedure, and first effects.

	*N*	%
Did s/he get a COVID-19 vaccination?		
No	179	22.6
Yes, mRNA vaccine	485	61.2
Yes, vector vaccine	127	16.0
Refusal to answer	2	0.3
Vaccination point		
Workplace	68	8.6
University	502	63.3
Other	42	5.3
Vaccine adverse effects		
No	225	36.8
Yes	378	61.8
Refusal to answer	9	1.4
Willingness to get a vaccine		
Definitely no	25	13.8
Rather no	34	18.8
Haven’t decided yet	35	19.3
Definitely yes	24	13.3
Rather yes	44	24.3
I cannot get vaccinated due to health reasons	19	10.5

**Table 3:** Analysis of the potential factors that could influence the decision for or against vaccination.

	Definitely No or Rather No		No Decision		Rather Yes or Definitely Yes		*χ* ^2^	*p*-Value *
	*N*	%	*N*	%	*N*	%		
**Year of university education**								
1	26	44.1	15	42.9	34	50.0	1.856	0.762
2	27	45.8	17	48.6	25	36.8		
3	6	10.2	3	8.6	9	13.2		
Sex								
Female	56	94.9	31	88.6	61	89.7	4.824	0.306
Male	3	5.1	2	5.7	6	8.8		
Refusal to answer	0	0.0	2	5.7	1	1.5		
Residence								
alone	7	11.9	3	8.6	10	14.7	2.475	0.649
With relatives/family/friends (excl. seniors)	43	72.9	25	71.4	42	61.8		
With relatives/family/friends (incl. seniors)	9	15.3	7	20.0	16	23.5		
Residence with a person from the COVID-19 risk group								
No	46	78.0	27	77.1	41	60.3	5.714	0.057
Yes	13	22.0	8	22.9	27	39.7		
Vaccination against flu								
Every season	3	5.1	2	5.7	1	1.5	4.961	0.291
Irregularly	7	11.9	6	17.1	17	25.0		
I do not vaccinate	49	83.1	27	77.1	50	73.5		
Developing COVID-19								
Yes (severe infection symptoms)	4	6.8	2	5.7	4	5.9	3.254	0.975
Yes (mild infection symptoms)	5	8.5	4	11.4	8	11.8		
Yes (no symptoms of infection)	2	3.4	0	0.0	1	1.5		
Probably (no test confirmation)	18	30.5	11	31.4	18	26.5		
No	19	32.2	12	34.3	20	29.4		
Don’t know myself	11	18.6	6	17.1	17	25.0		
Developing COVID-19 from closest environment								
Yes, severe or very severe	10	16.9	8	22.9	16	23.5	5.113	0.529
Yes, but rather mild	36	61.0	19	54.3	30	44.1		
No	11	18.6	5	14.3	16	23.5		
Don’t know	2	3.4	3	8.6	6	8.8		

* Chi-squared test.

## References

[1] Lurie N., Saville M., Hatchett R., Halton J. (2020). Developing Covid-19 Vaccines at Pandemic Speed. N. Engl. J. Med..

[2] Lamb Y.N. (2021). BNT162b2 mRNA COVID-19 Vaccine: First Approval. Drugs.

[3] WHO (2021). Status of COVID-19 Vaccines within WHO EUL/PQ Evaluation Process Manufacturer Name of Vaccine NRA of Record Platform EOI Accepted Pre-Submission Meeting Held Dossier Accepted for Review.

[4] Mathieu E., Ritchie H., Ortiz-Ospina E., Roser M., Hasell J., Appel C., Giattino C., Rodés-Guirao L. (2021). A global database of COVID-19 vaccinations. Nat. Hum. Behav..

[5] Raport Szczepień Przeciwko COVID-19—Szczepienie Przeciwko COVID-19—Portal Gov.pl. https://www.gov.pl/web/szczepimysie/raport-szczepien-przeciwko-covid-19.

[6] Szczepienia Personelu Medycznego—Szczepienie Przeciwko COVID-19—Portal Gov.pl. https://www.gov.pl/web/szczepimysie/informacje-dla-personelu-medycznego.

[7] Scholz N. (2021). Covid-19 Vaccination Campaigns: The Public Dimension.

[8] Petravić L., Arh R., Gabrovec T., Jazbec L., Rupčić N., Starešinič N., Zorman L., Pretnar A., Srakar A., Zwitter M. (2021). Factors affecting attitudes towards covid-19 vaccination: An online survey in Slovenia. Vaccines.

[9] Galanis P.A., Vraka I., Fragkou D., Bilali A., Kaitelidou D. (2020). Intention of health care workers to accept COVID-19 vaccination and related factors: A systematic review and meta-analysis. medRxiv.

[10] Rzymski P., Zeyland J., Poniedziałek B., Małecka I., Wysocki J. (2021). The Perception and Attitudes toward COVID-19 Vaccines: A Cross-Sectional Study in Poland. Vaccines.

[11] Dodd R.H., Pickles K., Nickel B., Cvejic E., Ayre J., Batcup C., Bonner C., Copp T., Cornell S., Dakin T. (2021). Concerns and motivations about COVID-19 vaccination. Lancet Infect. Dis..

[12] Porcelli A.J., Delgado M.R. (2017). Stress and decision making: Effects on valuation, learning, and risk-taking. Curr. Opin. Behav. Sci..

[13] Murphy J., Vallières F., Bentall R.P., Shevlin M., McBride O., Hartman T.K., McKay R., Bennett K., Mason L., Gibson-Miller J. (2021). Psychological characteristics associated with COVID-19 vaccine hesitancy and resistance in Ireland and the United Kingdom. Nat. Commun..

[14] Reyna V.F. (2012). A new intuitionism: Meaning, memory, and development in fuzzy-trace theory. Judgm. Decis. Mak..

[15] Sallam M. (2021). Covid-19 vaccine hesitancy worldwide: A concise systematic review of vaccine acceptance rates. Vaccines.

[16] Manning M.L., Gerolamo A.M., Marino M.A., Hanson-Zalot M.E., Pogorzelska-Maziarz M. (2021). COVID-19 vaccination readiness among nurse faculty and student nurses. Nurs. Outlook.

[17] Patelarou E., Galanis P., Mechili E.A., Argyriadi A., Argyriadis A., Asimakopoulou E., Brokaj S., Bucaj J., Carmona-Torres J.M., Cobo-Cuenca A.I. (2021). Factors influencing nursing students’ intention to accept COVID-19 vaccination–A pooled analysis of seven countries. medRxiv.

[18] Saied S.M., Saied E.M., Kabbash I.A., Abdo S.A.E.F. (2021). Vaccine hesitancy: Beliefs and barriers associated with COVID-19 vaccination among Egyptian medical students. J. Med. Virol..

[19] Lucia V.C., Kelekar A., Afonso N.M. (2020). COVID-19 vaccine hesitancy among medical students. J. Public Health.

[20] Mascarenhas A.K., Lucia V.C., Kelekar A., Afonso N.M. (2021). Dental students’ attitudes and hesitancy toward COVID-19 vaccine. J. Dent. Educ..

[21] Wawrzuta D., Jaworski M., Gotlib J., Panczyk M. (2021). What Arguments against COVID-19 Vaccines Run on Facebook in Poland: Content Analysis of Comments. Vaccines.

[22] Hlatshwako T.G., Shah S.J., Kosana P., Adebayo E., Hendriks J., Larsson E.C., Hensel D.J., Erausquin J.T., Marks M., Michielsen K. (2021). Online health survey research during COVID-19. Lancet Digit. Health.

[23] Geldsetzer P. (2020). Use of rapid online surveys to assess people’s perceptions during infectious disease outbreaks: A Cross-sectional Survey on COVID-19. J. Med. Internet Res..

[24] Kecojevic A., Basch C.H., Sullivan M., Chen Y.-T., Davi N.K. (2021). COVID-19 Vaccination and Intention to Vaccinate Among a Sample of College Students in New Jersey. J. Community Health.

[25] Gohel K.H., Patel P.B., Shah P.M., Patel J.R., Pandit N., Raut A. (2021). Knowledge and perceptions about COVID-19 among the medical and allied health science students in India: An online cross-sectional survey. Clin. Epidemiol. Glob. Health.

[26] Ikhlaq A., Bint-E-riaz H., Bashir I., Ijaz F. (2020). Awareness and attitude of undergraduate medical students towards 2019-novel corona virus. Pakistan J. Med. Sci..

[27] Mojally M., Al-Hindi Y. (2020). Knowledge and attitudes towards the novel coronavirus (COVID-19) among health care college students in Makkah, Saudi Arabia. Med. Sci..

[28] Wang P.-W., Ahorsu D.K., Lin C.-Y., Chen I.-H., Yen C.-F., Kuo Y.-J., Griffiths M.D., Pakpour A.H. (2021). Motivation to Have COVID-19 Vaccination Explained Using an Extended Protection Motivation Theory among University Students in China: The Role of Information Sources. Vaccines.

[29] Najbardziej Poważane Zawody przez Polaków w 2021. https://swresearch.pl/news/najbardziej-powazane-zawody-przez-polakow-w-2021.

[30] Rozporządzenie Ministra Zdrowia z dnia 9 kwietnia 2021 r. w Sprawie Kwalifikacji Osób Przeprowadzających Badania Kwalifikacyjne i Szczepienia Ochronne Przeciwko COVID-19. https://isap.sejm.gov.pl/isap.nsf/DocDetails.xsp?id=WDU20210000668.

[31] Yuan T., Liu H., Li X.D., Liu H.R. (2020). Factors affecting infection control behaviors to prevent COVID-19: An online survey of nursing students in Anhui, China in March and April 2020. Med. Sci. Monit..

[32] Bai W., Cai H., Liu S., Liu H., Qi H., Chen X., Liu R., Cheung T., Su Z., Ng C.H. (2021). Attitudes toward COVID-19 vaccines in Chinese college students. Int. J. Biol. Sci..

[33] CBOS News. https://www.cbos.pl/PL/publikacje/news/2021/04/newsletter.php.

[34] Tempski P., Arantes-Costa F.M., Kobayasi R., Siqueira M.A.M., Torsani M.B., Amaro B.Q.R.C., Nascimento M.E.F.M., Siqueira S.L., Santos I.S., Martins M.A. (2021). Medical students’ perceptions and motivations during the COVID-19 pandemic. PLoS ONE.

[35] Romer D., Jamieson K.H. (2021). Patterns of Media Use, Strength of Belief in COVID-19 Conspiracy Theories, and the Prevention of COVID-19 From March to July 2020 in the United States: Survey Study. J. Med. Internet Res..

